# Aldehyde dehydrogenase expression in *Metaphire posthuma* as a bioindicator to monitor heavy metal pollution in soil

**DOI:** 10.1186/s13104-016-2297-7

**Published:** 2016-11-21

**Authors:** Raju Panday, Padam Shekhar Bhatt, Tribikram Bhattarai, Kumudini Shakya, Lakshmaiah Sreerama

**Affiliations:** 1 Central Department of Biotechnology, Tribhuvan University, Kathmandu, Nepal; 2 Department of Botany, Amrit Science Campus, Lainchaur, Kathmandu, Nepal; 3 Department of Chemistry and Biochemistry, St. Cloud State University, St. Cloud, MN USA; 4 Department of Chemistry and Earth Sciences, Qatar University, Doha, Qatar

**Keywords:** *Metaphire posthuma*, Aldehyde dehydrogenase, Heavy metals, Lead (Pb), Cadmium (Cd), Bioindicators

## Abstract

**Background:**

Soil contamination and associated pollution plays a detrimental role in soil flora and fauna. Soil is processed and remodeled by subterranean earthworms, accordingly are referred to as soil chemical engineers. These worms, besides processing carbon and nitrogen, serve as minors for processing metals. In heavy metal contaminated soils, they accumulate heavy metals, which in turn cause altered gene expression, including aldehyde dehydrogenase (ALDH) enzymes. This study explores the possibility of ALDH expression in earthworms as a novel biomarker for the heavy metal contamination of soil.

**Results:**

Earthworms cultured in contaminated soils accumulated significantly higher levels of Pb and Cd. Similarly, significantly higher levels of ALDH enzyme activities were observed in earthworms cultured in soils contaminated with Pb and Cd. The ALDH activity was found to be highest in worms cultured in 5 ppm heavy metal contaminated soils. Although, ALDH activities decreased as the heavy metal concentration in soil increased, they were significantly higher when compared to control worms cultured in uncontaminated soils. The accumulation of heavy metal in earthworms measured after 28 days decreased as the heavy metal concentration in soil increased.

**Conclusions:**

Levels of ALDH expression correlated with total Pb and Cd concentration in the earthworm tissue. This study showed that the ALDH activity in earthworms could potentially be used as a biomarker to show heavy metal pollution in soil.

## Background

Soil is processed and remodeled by subterranean earthworms, accordingly they are referred to as soil chemical engineers. These worms, besides processing carbon and nitrogen, serve as minors for processing metals, including heavy metals in contaminated soils [1, 2]. Accordingly, earthworms are most commonly examined organisms for evaluating the uptake of soil contaminants. Earthworms have been shown to accumulate relatively high concentrations of heavy metals by either active or passive uptake mechanisms [1, 2]. Heavy metals such as lead (Pb) and cadmium (Cd) induce oxidative stress in cells by rendering cell’s major sulfhydryl reserves such as glutathione (GSH) inactive [3, 4]. These disturbances in the GSH levels generate free radicals which react with double bonds in membrane lipids resulting in an increase in lipid peroxidation [5]. This further promotes the mitochondrial respiration resulting in further increase in free radicals inside the cells. More than 200 highly reactive species, including malondialdehyde and 4-hydroxynonenal are known to be generated by the lipid peroxidation [6–8]. These toxic aldehydes are neutralized via the expression of a group of enzymes known as aldehyde dehydrogenases (ALDHs). ALDHs catalyze the conversion of aldehydes to their corresponding acids in an NAD(P)^+^-dependent irreversible reaction [9, 10]. With the metal accumulation inside worm tissues, it is assumed that this would induce expression of stress responsive proteins. Nematodes, e.g., *C elegans,* have been shown to express a variety of ALDHs in response to oxidative stress. [11]. Similarly, it is already known that exposure to sub-lethal concentrations of cadmium leads to over expression of a Cd- specific ALDH protein in *Enchytraeus buchholzi* [12] as well as cultured human tumor cells [13].

In the present study, changes in ALDH expression levels and heavy metal accumulation in the earthworm, *Metaphire posthuma*, were studied by growing them in soil spiked with different levels of lead and cadmium. Alongside, the possibility of using ALDH activity in earthworm as a biomarker for heavy metal contamination in soil was also assessed.

## Methods

### Earthworms and the soil substrate

Adult *Metaphire posthuma* earthworms (n = 120, mean weight 452.5 ± 63.5 mg and body length 865 ± 322 mm) were collected from the same soil that was used as control. Uncontaminated soil was collected from nearby the earthworm collection site. Background lead and cadmium levels of soil and earthworm were quantified by Atomic absorption spectrometry (AAS) (Table 1). The soil used possessed normal characteristic, with the exception that its moisture content and organic content were low. Accordingly, the moisture content of the soil in the trays was adjusted and maintained between 65 and 75%, and the pH was maintained between 5.5 and 6.7 during the exposure period. The green house and soil temperature varied daily from 26 to 30 and 22 to 25 °C during the study period respectively.

**Table 1:** Soil parameters of experimental soil used for the purpose of earthworm culture

Soil parameters	Values ± SD*
pH	6.22 ± 0.10
Organic content (%)	1.71 ± 0.04
Nitrogen (%)	0.09 ± 0.04
Potassium (mg/m^2^)	7.35 ± 1.26
Phosphorus (mg/m^2^)	0.221 ± 0.05
Moisture (%)	29 ± 1.70
Lead (ppm)	2.14 ± 0.08
Cadmium (ppm)	1.41 ± 0.01

*  Values are mean of three independent measurements

### Soil spiking and earthworm culture

Soils spiked with Pb and Cd (5–20 mg/kg) was prepared separately in plastic trays, all in triplicates. In each tray, 3 kg soil was mixed with lead nitrate solution (1000 ppm) or cadmium chloride solution (1000 ppm) to achieve the desired Pb and Cd concentrations. Tray without heavy metal spiked in soil was also prepared as a control in triplicates. The moisture content in trays was maintained at 70% during experiment. In every tray (27 trays in total), about 20 mature earthworms were added and cultured in dark for 28 days at 20–30 °C in greenhouse according to ISO 11268-1 [14].

### Sample preparation and ALDH activity measurement

Earthworms were harvested from the exposure trays every week until the final week of heavy metal exposure. The harvested earthworms were first washed with deionized water, longitudinally cut to remove intestinal cast, tissues were suspended in 5 ml enzyme assay buffer (64 mM sodium pyrophosphate and 2 mM EDTA),and ground in a mortar and pestle. The tissue debris was removed by centrifugation (500×*g* for 7 min) at 4 °C. The supernatant of the tissue homogenates were transferred to a fresh eppendorf tube and assayed for ALDH activity as follows.

ALDH assay was performed according to Sreerama and Sladek [9]. Briefly, a typical reaction mixture contained 32 mM sodium pyrophosphate, 1 mM EDTA, 4 mM NAD, 5 mM glutathione and 0.1 mM Pyrazole with 100 μl of sample. Rate of reaction rate (ΔA/min) was measured by adding the substrate (4 mM acetaldehyde) in a UV–Vis spectrophotometer for 5 min at 340 nm. Final reaction rates (mIU/min/mg) were calculated by subtracting the gross rate of reaction. The gross rate of reaction (ΔA/min) and linear slope curve were calculated by the spectrophotometer. The value obtained in control was subtracted from the gross rate to obtain the net rate of reaction. The specific ALDH activity (mIU/mg protein) was finally determined by dividing the ALDH activity (mIU/ml) by the concentration of protein in test preparation (mg/ml). The protein concentration in the tissue homogenates was determined using Bradford assay [15].

### Sample preparation for heavy metal analysis

Heavy metal analysis was performed in three different samples: (i) control soil, (ii) earthworms before exposure, and (iii) earthworms after exposure using a Perkin Elmer 240FS AAS as described by Maboeta [16]. One gram dried sample were mixed with 10 ml concentrated HNO_3_ and incubated at 60 °C for 2 h. The samples were then digested for about 4 h at 180 °C till the dark reddish fumes faded out and then filtered through Whatman no. 40 filter paper. The final filtrate volume was adjusted to 25 ml by using deionized water and used for the heavy metal analysis by AAS.

### Statistical analysis

All the reported results were expressed as mean of three replicates and analyzed using SPSS v16 (Statistical Program for Social Sciences). One way ANOVA was used to analyze the significant difference between the treatments. Duncan multiple range test was also performed to test the homogeneity of the results.

## Results

### ALDH activities in earthworms

The specific ALDH activities (mIU/mg protein) were calculated for *Metaphire posthuma* every week for the study period (Table 2). Specific ALDH activities were highest in earthworms grown in all lead spiked soil with respect to that in earthworms from control soil (Table 2). Specific ALDH activity increased with time (from week 1 to week 3) in all treatments. During the study period, specific ALDH activity was found to be highest in earthworms grown in 5 ppm lead spiked soil. However, specific ALDH activity in first week samples for worms grown on 20 ppm treatment of Pb (117.2 mIU/mg protein) was not statistically significant (Duncan: P value = 0.612) than that of the control (119.52 mIU/mg protein). The ALDH activity decreased as the concentration of lead increased in soil in all three weeks samples.

**Table 2:** Aldehyde dehydrogenase levels in whole homogenates of earthworm, viz., *M. posthuma*, exposed to different heavy metal treatments for three consecutive weeks

Treatment	Concentration (ppm)	ALDH activity (mIU/mg protein)		
		Week 1	Week 2	Week 3
None (control)	Zero	119.7 ± 5.02	62.3 ± 3.3	85.3 ± 3.7
Lead	5	240.6 ± 4.4	832.6 ± 12.1	944.5 ± 5.9
	10	172.2 ± 5.9	305.7 ± 8.6	696.3 ± 8.4
	15	146.8 ± 3.5	268.4 ± 8.3	595.5 ± 8.9
	20	117.2 ± 8.4	237.3 ± 7.5	265.2 ± 3.1
Cadmium	5	452.5 ± 10.6	664.8 ± 11.9	1871.7 ± 6.7
	10	314.4 ± 6.5	498.6 ± 10.8	1132.7 ± 12.6
	15	288.7 ± 12.4	475.3 ± 7.8	563.5 ± 8.6
	20	218.2 ± 3.1	184.2 ± 5.1	348.8 ± 3.7

*  Values represent mean specific activity of ALDH (mIU/mg protein) ± SD; n = 3. Earthworm tissue homogenate (prepared as described in methods section) was used to measure the ALDH activity using acetaldehyde (4 mM) as substrate and NAD (4 mM) as cofactor

The effect of cadmium in ALDH activity was similar to the effect shown by lead i.e. the specific ALDH activity was higher in earthworms grown in cadmium spiked soil than those grown in control soil and the activity increased with time in all treatments. Highest ALDH activity was observed in worms grown in 5 ppm cadmium treatment, similar to the Pb treatment. The specific ALDH activity decreased with the increase in cadmium concentration in soil.

However, specific ALDH activities were found to be higher in cadmium treatments than in the lead treatments. This might suggest that the cadmium is generally more toxic than lead at similar concentrations.

Given the ALDH activities are higher in earthworm tissues in every treatment group of both lead and cadmium as compared to the corresponding control groups, the results reported herein support the possibility of using ALDH activities as a molecular marker for studying heavy metal pollution in soil.

### Heavy metal concentration in earthworms

**Fig. 1:**
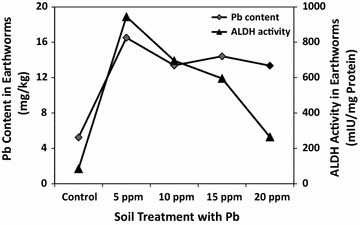
Correlation between tissue Pb concentrations (mg/kg) and tissue ALDH activities (mIU/mg protein)in earthworms (*M. posthuma)*grown in soils spiked (treated) with various concentrations of Pb

After the experiment period (28 days), the lead accumulation in earthworm tissues was significantly higher (Duncan: P = 0.05) in all treatments as compared to the control group (Fig. 1). The Pb content was found to be 2–3 folds greater in earthworms grown in Pb spiked soil than in control soil. The Pb content in worm tissues was lower in the present study than in the study carried out by Suthar and Singh [17]. This difference might be accounted for by the current use of a different earthworm species and the lower initial Pb content in the soil.

**Fig. 2:**
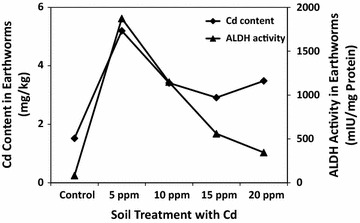
Correlation between tissue Cd concentrations (mg/kg) and tissue ALDH activities (mIU/mg protein) in earthworms (*M. posthuma)* grown in soils spiked (treated) with various concentrations of Cd

The Cd concentrations in earthworms after 28 days of exposure were significantly higher (Duncan: P = 0.05) as compared to the control group (Fig. 2). The Cd concentration in earthworms observed after 28 days of exposure were similar to those reported by Shahmansouri and coworkers [18]. The relationship between the ALDH activity and heavy metal accumulation by earthworms is shown in Figs. 1 and 2. The increase in ALDH activity in earthworm tissues was observed to be dependent on heavy metal concentration inside these tissues. Both the heavy metal accumulation and ALDH activities were the highest at 5 ppm concentration of either metal (Pb and Cd) in soil.

## Discussions

In this study, the specific activity of ALDH (mIU/mg total protein) in earthworm tissues increased with time in all treatments and increases in ALDH activity may be attributed to oxidative stress phenomenon caused by heavy metals. The above assertion is supported by the fact that (i) in *Caenorhabditis elegans*, the ALDHs are known to be induced by heavy metals for mitigating oxidative stress associated metabolism of lipid peroxidation-derived aldehydes [19] and (ii) ALDHs are also induced in cultured human cells in the presence of Cd and the induction of ALDHs in these cells is also attributed to oxidative stress [13].

In the present study both behavioral and physiochemical activities of the earthworm *M. posthuma* were considered. The stress response observed in the worms may be attributed to two possible mechanisms. First, the earthworms clearly avoid heavy metals by restricting themselves at the base of trays with stagnant physical activities. This kind of avoidance behavior may be of great significance for their survival. This has been observed by others in earthworm species *E. andrei* cultured in test soils contaminated with carbedazim and benzomyl at levels 10 ppm or higher [20]. Clearly, even if the toxic chemicals such as pesticides are low in concentration in the soil, their higher toxicity might switch off the regular active physiological activity in earthworms. In our study, the worms grown in soils containing 5 ppm heavy metal (Pb or Cd), the worms were found to be active consuming soil in greater amounts and borrowing through. On the other hand, at higher heavy metal (Pb or Cd) concentration in soil, worms reduced their feeding activity thereby accumulating lower concentrations of metals relative to 5 ppm metal treatment condition.

Secondly, earthworms are also known to defecate heavy metals to minimize their lethal effects [21]. The latter is accomplished by excess excretion of heavy metals relatively quickly and thus the tissues of the worms grown in soils with higher concentration of heavy metal would contain lower concentration of heavy metal. In our experiments this is, in fact, true, i.e., after the 28 days exposure, the tissues of the worms cultured at higher concentrations of heavy metals recorded lower concentrations of Pb and Cd as compared to the tissues of the worms grown in 5 ppm heavy metal. This is consistent with observations that increasing the concentration of Pb in soil did not result in similarly increased concentrations of Pb in *E. fetida* earthworm tissues [22]. In yet another study, Cd was found to be excreted in worm casts in low amounts even after short incubation period of 12 days [23]. Thus, it may be concluded that increasing the environmental concentration of heavy metals stimulates excretory mechanisms in earthworms [22, 23]. Accordingly, earthworms growing in soils with higher heavy metal concentrations excrete heavy metals accumulated in their tissues rapidly.

## Conclusions

This study confirms the idea that earthworms could potentially be used as a bioindicators to demonstrate heavy metal pollution in soil by measuring the expression levels of a stress responsive biomarker such as ALDHs. This is the study to illustrate the relationship between ALDH activity in *M. posthuma*, one of the commonly found earthworm species in Nepal, and heavy metal pollution in soil. Further molecular characterization of ALDHs and their role in heavy metal induced stress needs to be investigated.

## Abbreviations

GSH: glutathione
ALDH: aldehyde dehydrogenase
AAS: atomic absorption spectrometry
ISO: international standard organization
SPSS: statistical program for social sciences
